# Potential selection of antimony and methotrexate cross-resistance in *Leishmania infantum* circulating strains

**DOI:** 10.1371/journal.pntd.0012015

**Published:** 2024-02-29

**Authors:** Lorena Bernardo, Ana Victoria Ibarra-Meneses, Noelie Douanne, Audrey Corbeil, Jose Carlos Solana, Francis Beaudry, Eugenia Carrillo, Javier Moreno, Christopher Fernandez-Prada

**Affiliations:** 1 WHO Collaborating Centre for Leishmaniasis, Spanish National Center for Microbiology, Instituto de Salud Carlos III (ISCIII), Majadahonda, Madrid, Spain; 2 Centro de Investigación Biomédica en Red de Enfermedades Infecciosas (CIBERINFEC), ISCIII, Madrid, Spain; 3 Département de Pathologie et Microbiologie, Faculté de Médecine Vétérinaire, Université de Montréal, Saint-Hyacinthe, Quebec, Canada; 4 The Research Group on Infectious Diseases in Production Animals (GREMIP), Faculté de Médecine Vétérinaire, Université de Montréal, Saint-Hyacinthe, Quebec, Canada; 5 Département de Biomédecine, Faculté de Médecine Vétérinaire, Université de Montréal, Saint-Hyacinthe, Quebec, Canada; 6 Centre de recherche sur le cerveau et l’apprentissage (CIRCA), Université de Montréal, Montréal, Quebec, Canada; Fundacao Oswaldo Cruz, BRAZIL

## Abstract

**Background:**

Visceral leishmaniasis (VL) resolution depends on a wide range of factors, including the instauration of an effective treatment coupled to a functional host immune system. Patients with a depressed immune system, like the ones receiving methotrexate (MTX), are at higher risk of developing VL and refusing antileishmanial drugs. Moreover, the alarmingly growing levels of antimicrobial resistance, especially in endemic areas, contribute to the increasing the burden of this complex zoonotic disease.

**Principal findings:**

To understand the potential links between immunosuppressants and antileishmanial drugs, we have studied the interaction of antimony (Sb) and MTX in a *Leishmania infantum* reference strain (*Li*WT) and in two *L*. *infantum* clinical strains (*Li*FS-A and *Li*FS-B) naturally circulating in non-treated VL dogs in Spain. The *Li*FS-A strain was isolated before Sb treatment in a case that responded positively to the treatment, while the *Li*FS-B strain was recovered from a dog before Sb treatment, with the dog later relapsing after the treatment. Our results show that, exposure to Sb or MTX leads to an increase in the production of reactive oxygen species (ROS) in *Li*WT which correlates with a sensitive phenotype against both drugs in promastigotes and intracellular amastigotes. *Li*FS-A was sensitive against Sb but resistant against MTX, displaying high levels of protection against ROS when exposed to MTX. *Li*FS-B was resistant to both drugs. Evaluation of the melting proteomes of the two *Li*FS, in the presence and absence of Sb and MTX, showed a differential enrichment of direct and indirect targets for both drugs, including common and unique pathways.

**Conclusion:**

Our results show the potential selection of Sb-MTX cross-resistant parasites in the field, pointing to the possibility to undermine antileishmanial treatment of those patients being treated with immunosuppressant drugs in *Leishmania* endemic areas.

## Author summary

Visceral leishmaniasis (VL) is the most severe form of the disease caused by the parasite *Leishmania infantum*. Immunosuppressive conditions such as those generated using immunosuppressive treatments (i.e., methotrexate), to treat autoimmune diseases have increased the risk of developing severe complications linked to this parasitic disease, especially in endemic areas. Of note, treatment of VL in immunosuppressed patients is very challenging and frequently results in clinical relapse. For these reasons, it is capital to better understand any potential impact of the use of immunosuppressants on the antileishmanial effect of current drugs (i.e. antimonials) and their potential contribution to the emergence and spread of drug resistance. Here we report the first evidence of the potential co-selection of antimicrobial resistance between antimonials and methotrexate in *L*. *infantum* circulating strains. In addition to shedding some light on the causes of treatment failure and relapses in patients under methotrexate immunosuppression, this new knowledge could assist in the development of better immunosuppression strategies in endemic areas of leishmaniasis.

## Introduction

Leishmaniasis is a worldwide infectious disease caused by parasites of the genus *Leishmania* [1]. These parasites have two forms: the extracellular or promastigotes found in the sandfly vector and the intracellular or amastigotes found in the host cells [2]. Among the different clinical manifestations, visceral leishmaniasis (VL) is the most severe form of the disease, for which *Leishmania infantum* is the main causal agent [1]. VL is associated with elevated ranges of morbidity and mortality and 300,000 new cases are reported each year, where 95% of them are fatal if untreated [3,4]. In the absence of an effective vaccine, control of the disease is based on a very limited pharmacopeia with organic antimonials being one of the key drugs for VL treatment [5].

To bestow their antileishmanial activity, pentavalent antimonials (Sb^V^) must enter host infected cells and be reduced into the trivalent antimony (Sb^III^) [5,6]. Sb^III^ causes oxidative stress by increasing the concentration of reactive oxygen species (ROS), inducing DNA damage that leads apoptosis in the parasite [6,7]. Of note, nowadays, VL treatment is hampered, since the use of Sb is compromised due to *Leishmania* ability to develop and spread antimicrobial resistance, especially in endemic areas of the disease where these drugs have been continuously used in treating both human and canine patients [8,9]. Although metal resistance in *Leishmania* spp. is multifactorial, the main mechanism of Sb detoxification involves the ATP-binding cassette protein MRPA which binds to thiol-conjugated metals and promotes the exocytosis of these complexes outside the parasite [9–12]. In addition to an efficient pharmacological treatment, effective control of VL requires a protective Th1-type immune response by the host [13]. Consequently, immunosuppression represents the major individual risk factor to develop severe VL. This has been traditionally reported as an emerging problem in HIV co-infected patients [14,15]. Alarmingly, there is a recent increase in the number of VL cases among patients receiving immunosuppressant treatments to treat autoimmune diseases such as psoriasis, lupus erythematous or rheumatoid arthritis (RA) [16]. In these cases, VL treatment becomes more difficult and the risk of relapse increases [14,17].

Methotrexate (MTX) is, for more than 30 years, one of the most successful immunosuppressants for the control of inflammatory conditions (i.e., 60% of RA are currently on or have been on MTX). MTX is an antagonist of folic acid that interferes purine and pyrimidine synthesis by binding to dihydrofolate reductase (DHFR) and pteridine reductase 1 (PTR1) enzymes [18]. This results in a rapid depletion of intracellular levels of folates, which impairs DNA synthesis and leads to a decrease in cell proliferation [19]. *Leishmania* as well as other parasites are sensitive to MTX [20], although this drug is not used to treat leishmaniasis. However, as folates and pterins are essential for *Leishmania* development, these parasites can rapidly evolve resistance to MTX by increasing *dhfr-* and *ptr1*-gene dosage [21,22].

Whereas the mode of action and mechanism of drug resistance against Sb and MTX have widely explored in *Leishmania* in the past [6,23], there are no reports available on the potential effects on cross-tolerance or cross-resistance after exposure to any of these two different drugs. Here we report the first evidence of the potential co-selection of antimicrobial resistance between antimonial drugs and methotrexate in *L*. *infantum* circulating strains from untreated, naturally infected dogs. Moreover, the melting proteomes (meltome) of these strains, in the presence and absence of Sb and MTX, has identified differentially enriched direct and indirect targets for both drugs in different genetic backgrounds. This novel knowledge could bring some light into treatment failure and relapses occurring in patients under methotrexate immunosuppression, as well as to be the jumping-off point for tailoring better immunosuppression strategies in leishmaniasis endemic areas.

## Methods

### Parasites and cell lines

The study involved the use of different strains of *Leishmania* parasites: *Leishmania infantum* wild-type (*Li*WT) reference strain (MHOM/MA/67/ITMAP-263), as well as two *L*. *infantum* clinical isolates naturally circulating in non-treated VL dogs in Spain, namely *Li*FS-A (MCAN/ES/2004/LLM-1345) and *Li*FS-B (MCAN/ES/2005/LLM-1467). All strains were cultured in M199 medium (Wisent) supplemented with 10% heat-inactivated fetal bovine serum (FBS, Wisent) and 5 μg/mL of hemin (Millipore). The pH was maintained at 7.0, and the cultures were incubated at 25 °C. In addition, Bone Marrow-Derived Macrophages (BMDM) were cultured in DMEM medium supplemented with 10% FBS, 100 U/mL penicillin/streptomycin, and 20% L929 cell-conditioned medium.

### Drug-response assays in free-living promastigotes and intracellular amastigotes

The antileishmanial activity was assessed by monitoring the growth of non-exposed promastigotes for 72 hours at 25 °C in the presence of increasing concentrations of Sb (Potassium antimony tartrate sodium, Sigma) (0, 25, 50, 100, 150, 200, 300, 400 μM) or MTX (methotrexate, Sigma) (0, 10, 50, 100, 1000, 3000, 6000, 10000 nM). The optical density at 600 nm (A600) was measured using a Cytation 5 machine (Agilent, USA). Simultaneously, to investigate if exposure to one drug could induce cross-resistance or tolerance to the other, we subjected *Li*WT, *Li*FS-A, and *Li*FS-B promastigotes to the EC_50_ and the EC_90_ of either Sb or MTX (administered as single doses) over a period of five days. Following this, we performed a drug-response assay using the alternate drug (either Sb or MTX) on these ‘pre-exposed’ promastigotes utilizing the same spectrum of concentrations as previously described.

The intra-macrophage leishmanicidal activity of Sb (sodium stibogluconate, Calbiochem) and MTX was determined through *in vitro* infections, following our established protocols (9). Briefly, 2.5 × 10^5^ BMDM cells were seeded onto Ibidi 12-well chamber slides and maintained in complete DMEM medium. Metacyclic phase promastigotes of *Li*WT, *Li*FS-A, and *Li*FS-B were used at a BMDM to parasite ratio of 1:10 for the infection process. The cells were infected and allowed to incubate for 6 hours at 37°C with 5% CO2 in drug-free DMEM medium. After a 24-hours drug-free period, the medium was supplemented with increasing concentrations of MTX (0, 20, 50, 100, 200, 500 nM) or Sb (0, 10, 25, 50, 100, 200 μg/mL) for 5 days. To facilitate parasite visualization, the slides were fixed in methanol and stained with Diff-Quick solution. The number of infecting amastigotes per 100 cells was determined by examining 300 macrophages per triplicate assay and normalized to the untreated control.

In all these experiments, either targeting the promastigote or the amastigote stages, EC_50_ values were calculated based on dose-response curves analyzed by non-linear regression with GraphPad Prism 10.0 software (GraphPad Software, La Jolla California, USA). An average of at least three independent biological replicates run in triplicate was performed for each determination.

### Measurement of Reactive Oxygen Species (ROS) accumulation

Intracellular ROS accumulation was measured using the DCFDA dye (Invitrogen, USA) as previously described [12]. Briefly, 5 × 10^7^ mid-log *Li*WT, *Li*FS-A, and *Li*FS-B promastigotes were exposed to the EC_90_ of the drugs (i.e., Sb or MTX) for 48 hours in M199 medium at 25°C supplemented with 10% FBS and 5 μg/mL of hemin (pH 7.0). Parasites were washed twice in Hepes–NaCl (21 mM Hepes, 137 mM NaCl, 5 mM KCl, 0.7 mM Na_2_HPO_4_ 7H_2_O, 6 mM glucose, pH 7.4) and resuspended in 500 μL of Hepes–NaCl containing 25 μg/mL of H_2_DCFDA (Invitrogen, USA). Parasites were then incubated in the dark for 30 min and washed twice with Hepes–NaCl. After washing, 200 μL of the promastigote resuspension was analyzed with a Cytation 5 machine (Agilent, USA) at 485 nm excitation and 535 nm emission wavelengths. Fluorescence was normalized with the number of living parasites determined by propidium iodide (PI) staining and manual counting. Experiments were performed with at least three biological replicates from independent cultures, each of which included three technical replicates.

### Quantitative real-time RT-PCR

Total RNA was isolated from the three non-drug-exposed strains (*Li*WT, *Li*FS-A, and *Li*FS-B) using the RNeasy Mini Kit (Qiagen), following the manufacturer’s instructions, as has been described earlier [24]. Additionally, total RNA was extracted from MTX-exposed *Li*WT obtained during ‘pre-exposure’ experiments (S1 Fig). The cDNA was synthesized using the iScript Reverse Transcription Supermix (Bio-Rad) and amplified in the iTaq universal SYBR Green Supermix Kit (Bio-Rad) using a CFX Opus Real-Time PCR System (Bio-Rad). The expression levels of ATP-binding cassette protein MRPA (*LinJ*.*23*.*0290*; Fw: 5′-CGCATTATGCTGTGGTTCCG-3′; Rv: 5′-GTCGTACTCGCCCATCAGAG-3′), dihydrofolate reductase thymidylate synthase DHFR-TS (*LinJ*.*06*.*0890*; Fw: 5′-CGCATCATGAAGACGGGGAT-3′; Rv: 5′-TGAATGTCCTTGGCCAG-3′); argininosuccinate synthase ASS (*LinJ*.*23*.*0300*; Fw: 5′-CTTCTGAGGCTGTGCAACAC-3′; Rv: 5′-GATGCCCTTCTGGAACTGGA-3′) and pteridine reductase 1 PTR1 (*LinJ*.*23*.*0310*; Fw: 5′-TATACCATGGCCAAAGGGGC-3′; Rv: 5′-TGACGTACTTGGCCTTGGGA-3′) were derived from three technical and three biological replicates and were normalized to constitutively expressed mRNA encoding glyceraldehyde-3-phosphate dehydrogenase GAPDH (*LinJ*.*36*.*2480*; Fw: 5′-GTACACGGTGGAGGCTGTG-3′; Rv: 5′-CCCTTGATGTGGCCCTCGG-3′).

### Comparative meltome analysis using thermal proteomic profiling (TPP)

For TPP analysis, *Li*FS-A and *Li*FS-B were prepared following our previously described methods [25]. In brief, cultures of *Li*FS-A and *Li*FS-B isolates in the mid-log phase underwent multiple centrifugation steps. We conducted experiments with biological triplicates for each isolate. The resulting pellet was washed with PBS 1× (pH 7.4, Gibco, Life Technologies) and then resuspended in 5 mL of lysis buffer. The lysis buffer consisted of 50 mM mono-basic potassium phosphate, 50 mM di-basic potassium phosphate, 0.5 M EDTA, 1 M DTT, 10 mM tosyl-L-lysyl-chloromethane hydrochloride, 0.8% n-octyl-β-D-glucoside, and mini protease inhibitor cocktail (EDTA-free). To obtain sufficient protein, three freeze-thaw cycles were performed, followed by centrifugation at 20,000 g for 20 minutes at 4 °C. The protein yield required for the TPP experiment was 4 mg.

Once the lysate was obtained, drug-induced disruption and heat treatment were performed. Each lysate was divided into three subsamples: 100 μM Sb, 100 μM MTX, and a control (vehicle). For each condition, 250 μg of lysate (approximately 100 μL) was added to seven microcentrifuge tubes, with each tube representing a different temperature (37, 45, 50, 55, 60, 65, and 70 °C). The tubes were incubated for three minutes, followed by centrifugation at 20,000 g for 20 minutes at 4 °C to recover the soluble protein fraction. The soluble proteins were collected by precipitation using cold acetone and 50 mM tris-HCl, followed by alkylation with 40 mM 2-Iodoacetamide (IAA, Sigma) and digestion with a 1:20 trypsin solution for 24 hours. After incubation, the samples were labeled using a light (test samples) and heavy (internal standard; *L*. *infantum* WT maintained at 37 °C) dimethyl strategy and mixed for consecutive HPLC-MS/MS analysis using a duplex labeling approach. High-performance liquid chromatography (HPLC) was performed using a Thermo Scientific Vanquish FLEX UHPLC system (San Jose, USA) with gradient elution on a microbore column (particle size: 5 μm, Thermo Biobasic). The mobile phase, a mixture of acetonitrile and water containing 0.1% formic acid, was subjected to a linear gradient shift from 5:95 to 40:60 over a duration of 63 minutes. Detection in the positive ion mode was carried out using a Thermo Scientific Q Exactive Plus Orbitrap Mass Spectrometer, which was integrated with the UHPLC system. The TOP-10 Data Dependent Acquisition method was employed for this purpose. Rather than treating each replicate as an individual sample, we opted to pool the data from these replicates, thereby combining their results to form a single, comprehensive dataset for each condition. The data processing for the study was carried out using Thermo Proteome Discoverer (version 2.4), in combination with SEQUEST. The analysis involved a curated database with FASTA sequences from UniProt specific to *L*. *infantum* (TAXON ID 5671). Key settings included an MS^1^ tolerance of 10 ppm, MS^2^ mass tolerance of 0.02 Da for Orbitrap detection, and trypsin specificity with allowance for two missed cleavages. Fixed modifications included carbamidomethylation of cysteine and dimethylation of lysine and N-terminus, while oxidation of methionine was a variable modification. The minimum peptide length was set at six amino acids, excluding proteins identified by only one peptide. Protein quantification and comparative analysis were based on peak integration, using the average ion intensity of unique peptides to determine protein abundance. For normalization purposes, the protein abundance value at the lowest examined temperature (37°C) was set as the baseline, represented by a value of 1. The generated melting curves were inspected for a change in melting behavior following the formula described by Franken et al (2015) [26]. All melting curves were created using GraphPad Prism 10. The temperature resulting in a 50% of protein denaturalization was defined as the melting temperature (*T*_*m*_), which was used to calculate the cut-off value (*ΔT*_*m*_ = *T*_*m*_ drug–*T*_*m*_ control). Heat maps were generated through the Heat mapper webserver (www.heatmapper.ca/expression) using its protein expression plugin with average linkage as clustering method applied to rows and Euclidean as distance measurement method. The complete proteomics dataset is available in (S1 Data).

## Results

### *L*. *infantum* clinical isolates display different sensitivity profiles and enhanced ability to control oxidative stress in the presence of antimony and methotrexate

The drug-resistant profile of current *L*. *infantum* strains circulating in dogs presents a significant challenge in the treatment of both canine and human leishmaniasis, especially in pharmacologically immunosuppressed individuals. Several studies have reported an alarming increase in drug resistance, particularly to commonly used antileishmanial drugs such as Sb. In this context, we first evaluated the sensitivity profile of *Li*FS-A and *Li*FS-B, two clinical isolates recovered from non-treated, naturally infected dogs in Spain [27]. For comparison purposes, we included the *L*. *infantum* ITMAP-263 laboratory reference strain (*Li*WT), which is known to be sensitive to the different antileishmanials. As summarized in Table 1, clinical isolate *Li*FS-A showed similar levels of Sb sensitivity of those measured for the reference strain (75.35 *vs*. 68.24 μM Sb^III^ in promastigotes; and 68.71 *vs*. 99.35 μg/mL Sb^V^ in amastigotes). Of note, *Li*FS-B showed a clear resistant profile with EC_50_ values > 2.5-fold when compare with the *Li*WT reference strain (198.2 μM Sb^III^ in promastigotes; and > 200 μg/mL Sb^V^ in amastigotes). While there is no report in the literature of treating leishmaniasis with MTX, this drug is frequently used off label for the treatment of immune-mediated diseases, such as immune-mediated hemolytic anemia and immune-mediated polyarthritis in dogs. As expected, the *Li*WT strain showed a very sensitive phenotype against this drug in both promastigotes and amastigotes (1.03 and 0.34 μM, respectively). Conversely, both clinical isolates displayed high levels of resistance as both free and intracellular parasites (>500 and > 200 μM, respectively).

**Table 1:** EC_50_ values for methotrexate and antimony in *Leishmania* promastigotes and amastigotes calculated from concentration-response curves.

	EC_50_ (95% CI)	
	Methotrexate	Antimony*
***Li*WT**		
Promastigote	1.03 μM (0.80–1.26)	75.35 μM (70.65–80.35)
Amastigote	0.34 μM (0.29–0.42)	68.71 μg/mL (55.07–91.21)
***Li*FS-A**		
Promastigote	> 500 μM (N.A.)	68.24 μM (60.02–77.57)
Amastigote	> 200 μM (N.A.)	99.35 μg/mL (84.00–102.60)
***Li*FS-B**		
Promastigote	> 500 μM (N.A.)	198.2 μM (180.6–217.5 μM)
Amastigote	> 200 μM (N.A.)	> 200 μg/mL (N.A.)

* Promastigotes were subjected to experiments using trivalent Sb, while pentavalent Sb was employed for experiments involving amastigotes. N.A. = Not available

**Fig 1:**
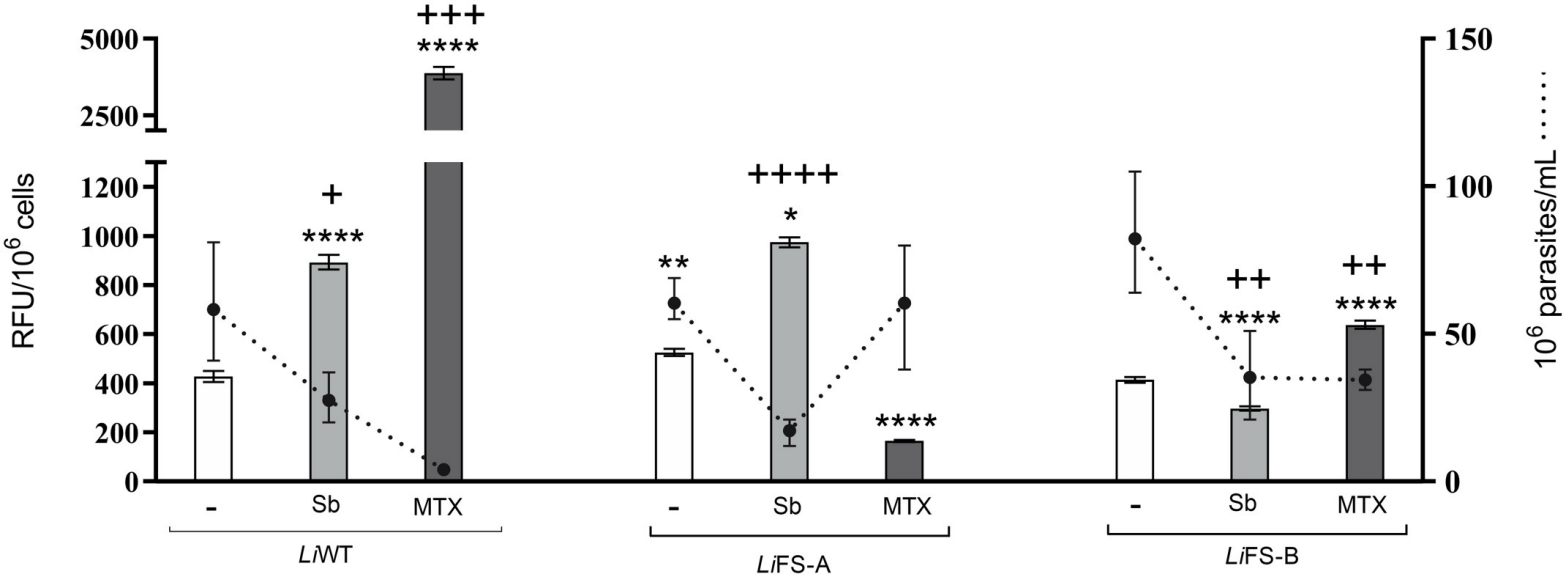
Evaluation of ROS accumulation and parasite survival in the absence and the presence of Sb and MTX. Measurement of drug-induced (Sb and MTX) ROS accumulation (DCFDA fluorescence; Cytation 5; ex/em 485/535 nm) in *L*. *infantum* WT and *Li*FS-A and *Li*FS-B clinical isolates. Graphs represents the number of viable promastigotes normalized to 10^6^ cells/mL (dotted line) and DCFDA fluorescence normalized to 10^6^ promastigotes (bars). Each data point represents the average ± SEM. Differences were statistically evaluated using an unpaired two-tailed t-test (*,^+^ p <0.05; **,^++^ p <0.01; ^+++^ p < 0.001; ****,^++++^ p < 0.0001).

Antimicrobial resistance mechanisms in *Leishmania* can involve alterations in drug targets, decreased drug uptake, increased drug efflux, and enhanced antioxidant defenses. To explore this last feature, we examined the impact of Sb and MTX on the ability of *Li*WT, *Li*FS-A and *Li*FS-B to control reactive oxygen species (ROS) accumulation (Fig 1). In this way, the three strains were exposed to the EC_90_ of Sb and MTX. DCFDA fluorescence emission and parasite survival rates were simultaneously measured. Sb and MTX induced major accumulation of ROS in *Li*WT (up to 892 and 3868 relative fluorescence units (RFU), respectively) after a 48-h exposure to the EC_90_ of Sb and MTX. This was coupled with a significant reduction in the presence of viable parasites, which was reduced by more than 53% and 94% when exposed to Sb and MTX, respectively. This is consistent with the antileishmanial effect previously described for these drugs [7,28]. Both *Li*FS-A and *Li*FS-B displayed similar basal levels of ROS (and similar to *Li*WT) in the absence of drug pressure. Exposure to Sb led to similar ROS and viability levels in *Li*FS-A when compared with the reference strain (⁓980 RFU), which is in agreement with its Sb-sensitive profile–as per determined in the drug-response assays. In contrast, *Li*FS-B exhibited approximately 2-fold lower ROS accumulation compared to *Li*WT and demonstrated better survival rate when exposed to Sb, providing additional evidence for its Sb-resistant phenotype. Both *Li*FS-A and *Li*FS-B exhibited reduced ROS accumulation and enhanced survival compared to *Li*WT when exposed to MTX. *Li*FS-A demonstrated approximately 20-fold lower ROS accumulation than *Li*WT and displayed a similar survival rate to the untreated control (approximately 100%). Conversely, *Li*FS-B accumulated around 20-fold less ROS than the reference strain but exhibited lower viability than the untreated control (approximately 50%). These findings further support the MTX-resistant phenotype observed in both isolates and suggest enhanced ability to control oxidative stress.

### Overexpression of key drug-resistance genes and ‘pre-exposure’ to Sb or MTX contribute to multidrug-resistance phenotypes

**Fig 2:**
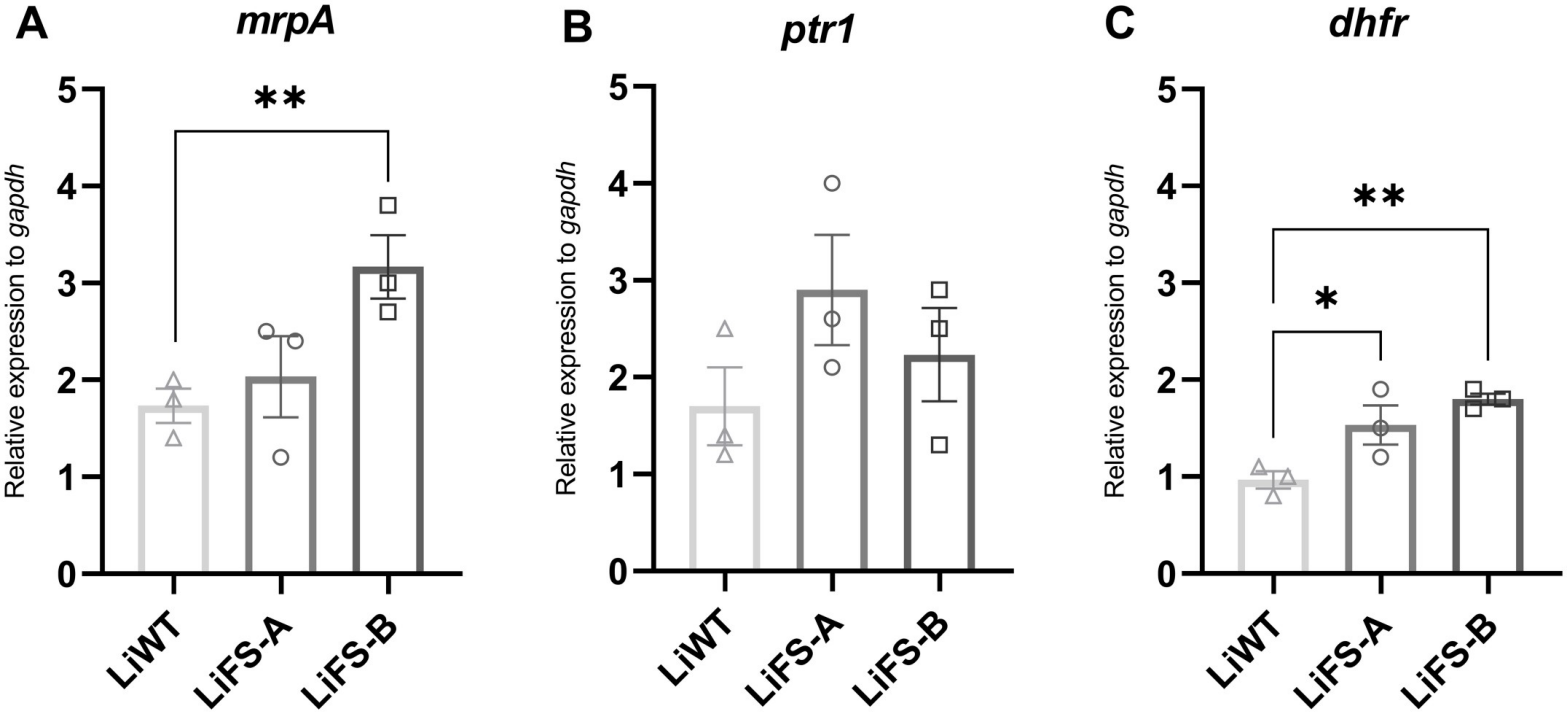
Normalized mRNA expression levels of *mrpA*, *ptr1* and *dhfr* in non-exposed parasites. mRNA expression levels of drug-resistance genes *mrpA* (A), *ptr1* (B) and *dhfr* (C) were determined by quantitative real-time RT-PCR in *Li*WT, *Li*FS-A and *Li*FS-B strains and normalized using *gapdh* as housekeeping gene. Results are derived from three biological replicates. Each data point represents the average ± SEM. Differences were statistically evaluated using an unpaired two-tailed t-test *p<0.05; ** p<0.01.

One of the most frequent mechanisms deployed by *Leishmania* parasites to overcome the action of Sb and MTX is upregulating the expression of drug targets and drug-resistance genes. Overexpression of the gene coding for an ABC-thiol transporter multidrug resistance protein A (*mrpA*) is frequently reported in Sb-resistant parasites, leading to the intracellular sequestration and subsequent elimination of Sb-thiol conjugates [9]. Likewise, MTX-resistance is associated with the overexpression of dihydrofolate reductase (*dhfr*) and pteridine reductase 1 (*ptr1*) genes, respectively, encoding the primary and secondary targets of MTX [21]. For that reason, we evaluated the expression of *mrpA gene*, or *dhfr* and *ptr1* genes, associated to Sb or MTX resistance, respectively, in non-exposed promastigotes. Our results illustrated a notable increase in *mrpA* expression in *Li*FS-B compared to both *Li*WT and *Li*FS-A (Fig 2A), providing further evidence for its classification as Sb-resistant (Table 1). Regarding MTX genes of resistance, no significant differences in the expression levels of *ptr1* were found (Fig 2B). Nonetheless, in *Li*FS-A, we observed a non-significant trend in the expression of *mrpA* and *ptr1*. Both clinical isolates showed a higher expression of the *dhfr* gene when compared with the reference strain *Li*WT (Fig 2C), which could contribute to the survival of these parasites in higher concentrations of MTX as previously reported [12,21].

**Fig 3:**
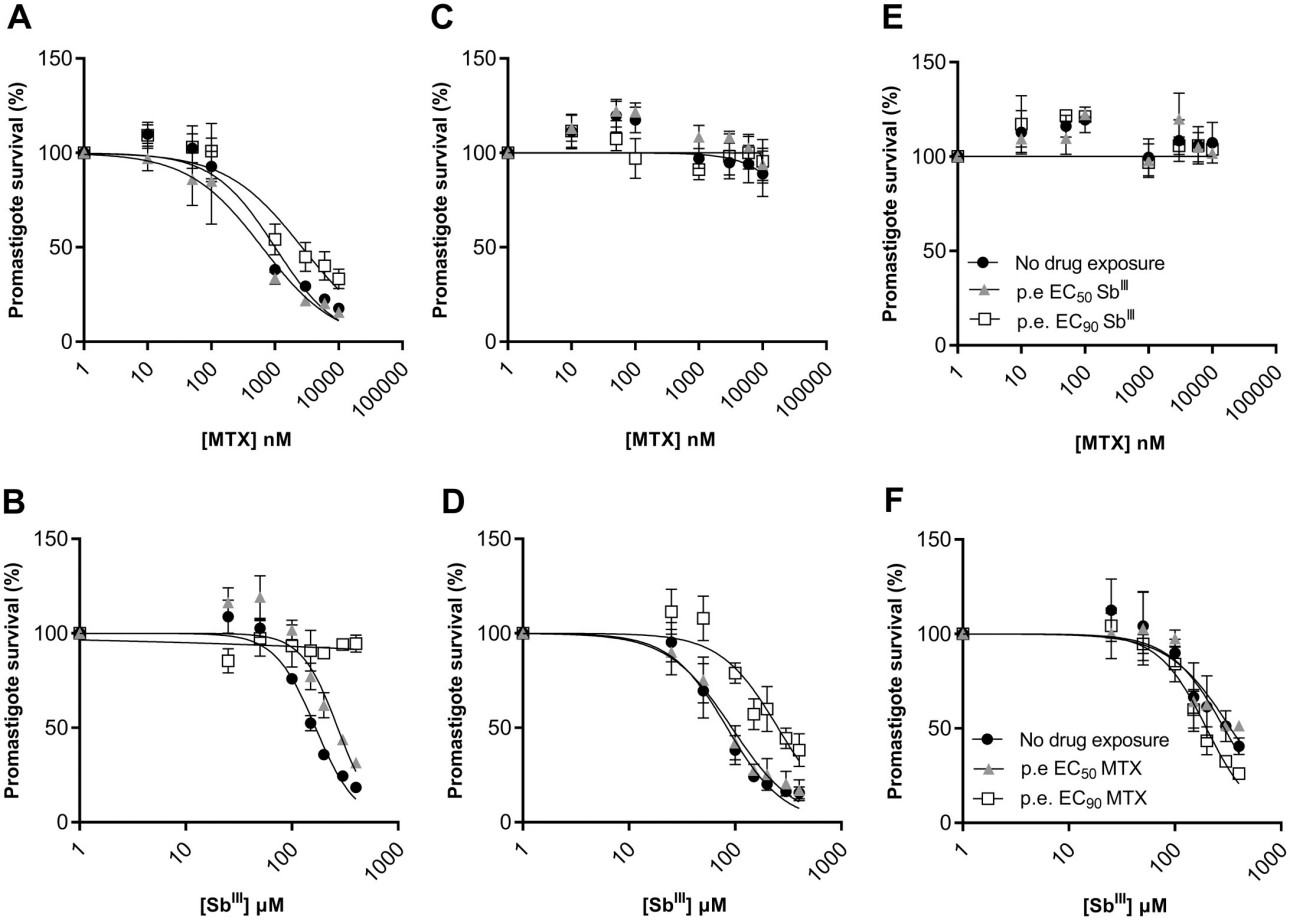
Phenotypic characterization of *L*. *infantum* WT reference strain and *Li*FS-A and *Li*FS-B clinical isolates after ‘pre-exposure’ to Sb and MTX. Five days after ‘pre-exposing’ *Li*WT **(A-B)**, *Li*FS-A **(C-D)**, *Li*FS-B **(E-F)** promastigotes to the EC_50_ and EC_90_ (previously calculated; Table 1) of Sb or MTX, parasites were submitted to increasing concentrations of MTX and Sb to evaluate potential changes in their phenotype against these drugs. EC_50_ values were calculated from concentration-response curves performed with biological triplicates after nonlinear fitting with GraphPad Prism 10 software.

Next, to further understand the potential effect of a pharmacological immunosuppression (i.e., induced by MTX) on the outcome of *L*. *infantum* treatment (i.e., induced by Sb), we evaluated the impact of a single-dose exposure (‘pre-exposure’ to EC_50_ or EC_90_ for 5 days) to MTX or Sb prior to characterizing these parasites in drug-response assays. As depicted in Fig 3A, ‘pre-exposure’ to Sb EC_90_ led to a significant reduction in MTX sensitivity in *Li*WT parasites (2.65-fold). Markedly, this phenomenon was bidirectional, and ‘pre-exposure’ to either the EC_50_ or EC_90_ of MTX resulted in a great decrease in the sensitivity of the *Li*WT reference strain to Sb (Fig 3B). The effect was maximal when exposing *Li*WT to MTX EC_90_ (Fig 3A), probably due to the rapid emergence of a subset of the population carrying amplifications of the H locus which contains *ptr1* but also *mrpA* [29]. This aspect was further investigated by evaluating the expression levels of *ptr1* and *mrpA* in *Li*WT pre-exposed to EC_90_ of MTX, along with argininosuccinate synthase (*ass*), a third gene within the H locus. As anticipated, the population that was recovered exhibited a significant increase in the mRNA expression levels of these three genes (S1 Fig). ‘Pre-exposure’ to MTX in the Sb-sensitive *Li*FS-A strain led to a significant decrease in its levels of sensitivity (up to 3.01-fold) against antimonial drugs (Fig 3D). This effect was not observed in the *Li*FS-B strain which was already resistant to Sb (Fig 3F). As expected, no measurable effect was detected when exposing *Li*FS-A and *Li*FS-B clinical isolates to Sb, as both are highly resistant to MTX (Fig 3C and 3E). The findings indicate that cross-resistance between antimony and methotrexate can manifest equally, regardless of the drug administered first. Additionally, the results suggest different multidrug-resistance phenotypes which a swift and transient emergence of Sb-resistant parasites upon exposure to MTX.

### Clinical isolates’ melting proteomics reveals different protein interactions with the drugs pointing to different mechanisms of drug resistance

The mechanisms underlying antimony resistance have been extensively described in laboratory lines of *Leishmania*. However, there may be differences between these mechanisms and those operating in *Leishmania* circulating isolates [30]. To map Sb and MTX potential targets (both direct and indirect) in *Li*FS-A and *Li*FS-B clinical isolates, we used a powerful multiplexed, quantitative mass spectrometry-based proteomics approach named Thermal Proteomic Profiling (TPP), which enables monitoring the melting profile of thousands of expressed soluble proteins in drug-sensitive and drug resistant *L*. *infantum* parasites, in the presence (or absence) of any antileishmanial drug [25]. As previously described, in our TPP approach, we used a fixed concentration of drug (100 μM SB or MTX) for the induction of drug-driven disruption and seven different temperatures for the temperature range (37–70 °C) [25].

**Fig 4:**
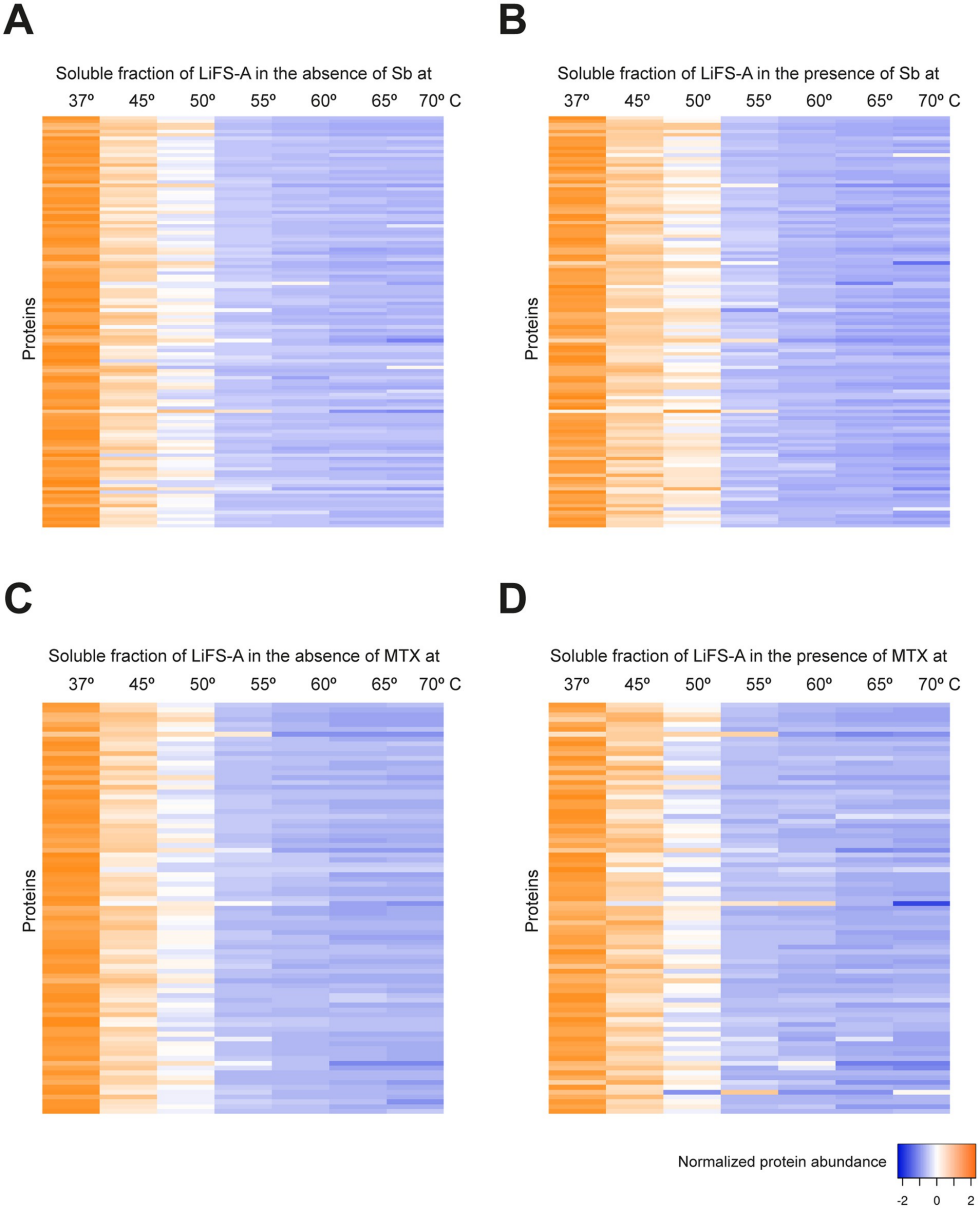
Heat map representation (row Z-score) of the general thermal stability of *Li*FS-A soluble protein cell extracts. Normalized protein abundance of *Li*FS-A proteins for which full melting curves were acquired in the absence (**A**) or in the presence (**B**) of 100 μM Sb (118 proteins) and in the absence (**C**) or in the presence (**D**) of 100 μM MTX (84 proteins). Color range depicts the relative protein abundance of the soluble fractions at different temperatures. Heat maps were generated through the Heat mapper webserver (www.heatmapper.ca/expression) using its protein expression plugin with average linkage as clustering method applied to rows and Euclidean as distance measurement method.

We first measured the impact of Sb and MTX on the thermal stability of the soluble proteins of *Li*FS-A strain (Sb-sensitive and MTX-resistant; Table 1). We obtained and analyzed quantitative data to determine the thermal stability of 1147 soluble proteins (S1 Data). Out of these proteins, 118 exhibited variations in their thermal stability and their Δ*T*_*m*_ was positive (Δ*T*_*m*_ > 0) (Table 2). As depicted in Fig 4A and 4B, these proteins demonstrated enhanced stability at lower temperatures, specifically between 37 and 50 °C. Noteworthy proteins in this category included a mitochondrial elongation factor (E9AGQ3), a putative iron-sulfur reiske protein (A4IB55), a calmodulin-like protein (A4IBS7), as well as various ribosomal proteins (L6, L12, L13, L23, S20, L34, S6, S13, S4, L18, and S9). Conversely, in our investigation of the interaction between the *Li*FS-A strain and MTX, we identified 1158 soluble proteins. Among these proteins, we obtained melting curve profiles in the presence of MTX for 84 of them (Fig 4C and 4D). Table 2 provides a summary of the proteins identified in this analysis, including PTR1 (A4I067), two ribosomal proteins (A4HS42 and A0A6L0XG31), an amidohydrolase (A4I5G9), the cytochrome C1 mitochondrial protein (A4HT63), and an oligopeptidase b (A4HTZ8), among others.

**Table 2:** Summary of proteins identified in Sb-treated and MTX-treated *Li*FS-A, demonstrating a positive temperature shift. Proteins that are common between the strains *Li*FS-A and *Li*FS-B are highlighted in bold for easy identification.

**Sb**					
**Accession**	**Gene ID**	**Description**	***T***_***m***_ **(°C)**		***ΔT***_***m***_ **(°C)**
			**+ Sb**	**- Sb**	
E9AGQ3	*LINF 180012600*	Elongation factor Tu–mitochondrial—putative	49.24	40.46	8.78
A4IB55	*LINF 350020400*	Putative reiske iron-sulfur protein	50.99	42.3	8.69
A4IBS7	*LINF 350044300*	Calmodulin-like protein	48.18	40.38	7.8
A4HWJ8	*LINF 150018200*	60S ribosomal protein L6	59.53	52.2	7.33
Q9N9V3	*LINF 040012600*	Putative ribosomal protein L10	54.86	47.69	7.17
A4IB12	*LINF 350016600*	NADH-dependent fumarate reductase—putative	50.31	43.54	6.77
A4HXG5	*LINF 170015800*	META domain containing protein	50.29	43.85	6.44
A4I4Y2	*LINF 290036400*	40S ribosomal protein S19-like protein	50.32	44.58	5.74
A4HSV3	*LINF 060011800*	Putative Phosphatase /Protein of uncharacterized function DUF89—putative	51.05	45.37	5.68
A4I397	*LINF 280007300*	Glycerol-3-phosphate dehydrogenase	49.03	43.99	5.04
A4I9C1	*LINF 330037300*	Hypothetical protein—conserved	49.12	44.14	4.98
E9AHW0	*LINF 240028300*	60S ribosomal protein L12—putative	50.04	45.22	4.82
A4I7G5	*LINF 320005100*	Nuclear segregation protein—putative	51.85	47.62	4.23
A4HT63	*LINF 070005600*	Cytochrome c1—heme protein—mitochondrial—putative	47.88	43.92	3.96
A4I8D8	*LINF 340035600*	Putative ribosomal protein L3	52.5	48.63	3.87
A4HRY4	*LINF 030014900*	Eukaryotic initiation factor 2a—putative	46.81	43.05	3.76
A4I0S4	*LINF 240013700*	Triosephosphate isomerase	50.55	46.82	3.73
**E9AG68**	***LINF 060011300***	**60S ribosomal protein L23a—putative**	**49.89**	**46.23**	**3.66**
A4IAZ4	*LINF 350014400*	Aldose 1-epimerase—putative	43.96	40.36	3.6
A4I3X3	*LINF 280031300*	2-oxoglutarate dehydrogenase—E2 component—dihydrolipoamide succinyltransferase—putative	51.3	47.72	3.58
A4I116	*LINF 240026900*	40S ribosomal protein S8	50.72	47.22	3.5
A4HRV7	*LINF 030011900*	DEAD/DEAH box helicase /Type III restriction enzyme—res subunit—putative	43.84	40.35	3.49
A4IAL2	*LINF 340052200*	1 -2-Dihydroxy-3-keto-5 -methylthiopentene dioxygenase—putative	48.2	44.92	3.28
A4HVL5	*LINF 130013600*	Mitochondrial processing peptidase alpha subunit—putative	45.92	42.65	3.27
E9AHH9	*LINF 280015700*	Putative ribosomal protein S20	46.33	43.16	3.17
A4HRT1	*LINF 030006800*	Delta-1-pyrroline-5-carboxylate dehydrogenase—putative	46.58	43.45	3.13
E9AGD4	*LINF 120009800*	Hypothetical protein—conserved	49.22	46.12	3.1
A4HY10	*LINF 180019400*	60S ribosomal protein L34—putative	51.09	48.02	3.07
A4HZP2	*LINF 220006400*	Hypothetical protein—conserved	55.06	52.12	2.94
Q6RYT3	*LINF 290017500*	Ttryparedoxin 1—putative	55.22	52.28	2.94
A0A381MG06	*LINF 190005300*	Histone H2B	52.17	49.3	2.87
**A4HUB4**	***LINF 100005600***	**Putative ribosomal protein l35a**	**49.37**	**46.6**	**2.77**
**A4HZF8**	***LINF 210024300***	**ATP-dependent RNA helicase SUB2—putative**	**48.35**	**45.73**	**2.62**
A4I2F5	*LINF 260028200*	Nitrilase—putative	47.92	45.3	2.62
A4HZI4	*LINF 350025100*	40S ribosomal protein S6	48.65	46.1	2.55
A4HY61	*LINF 330041600*	40S ribosomal protein S13—putative	49.99	47.46	2.53
A0A6L0XIU2	*LINF 290021400*	Nodulin-like—putative	47.24	44.71	2.53
A4HVQ1	*LINF 130017300*	Putative 40S ribosomal protein S4	48.59	46.23	2.36
A4I0D8	*LINF 230016800*	Alcohol dehydrogenase—zinc-containing-like protein	48.05	45.69	2.36
Q9BHZ6	*LINF 090016000*	Elongation factor-1 gamma	46.46	44.15	2.31
A0A6L0WKL6	*LINF 100016800*	Histone H3—putative	50.44	48.2	2.24
Q9N9V8	*LINF 300042300*	Ribosomal protein L15	47.17	44.96	2.21
A4HUC9	*LINF 100007300*	Nucleolar protein 56—putative	46.63	44.44	2.19
**A4I4W0**	***LINF 290032300***	**60S ribosomal protein L13—putative**	**49.06**	**46.91**	**2.15**
A4HVI5	*LINF 130010500*	60S ribosomal protein L18—putative	49.55	47.48	2.07
A4HT92	*LINF 070012400*	40S ribosomal protein S9—putative	48.19	46.17	2.02
A4I784	*LINF 310036600*	ADP-ribosylation factor—putative	47.24	45.24	2
Q95U89	*LINF 230005400*	Peroxidoxin	47.38	45.39	1.99
**A4HWV9**	***LINF 160010900***	**Core histone H2A/H2B/H3/H4/Histone-like transcription factor (CBF/NF-Y) and archaeal histone—putative**	**50.68**	**48.73**	**1.95**
**A4I5X6**	***LINF 300035000***	**Glyceraldehyde-3-phosphate dehydrogenase**	**48.33**	**46.38**	**1.95**
**A4I7K4**	***LINF 320009100***	**ATP-dependent RNA helicase eIF4A**	**48.34**	**46.41**	**1.93**
**Q4VT69**	***LINF 210007900***	**Hexokinase—putative**	**50.67**	**48.77**	**1.9**
A4I115	*LINF 240026800*	Transketolase	51.79	49.91	1.88
A0A6L0XZX8	*LINF 350009300*	40S ribosomal protein S3a	47.89	46.04	1.85
A0A381M920	*LINF 010012800*	ATP-dependent RNA helicase eIF4A	47.36	45.54	1.82
A4ICM4	*LINF 360016500*	Putative ribosomal protein L24	48.31	46.52	1.79
A4HVS0	***LINF 130021300***	**Ubiquitin-conjugating enzyme-like protein**	**45.3**	**43.55**	**1.75**
E9AHZ7	*LINF 360078300*	Phosphoglycerate mutase (2,3-diphosphoglycerate-independent)	48.05	46.34	1.71
A4IA92	*LINF 340039400*	Alpha-keto-acid decarboxylase—putative	50.25	48.57	1.68
A4HSH2	*LINF 050010000*	ATP synthase F1—alpha subunit—putative	53.04	51.38	1.66
A4I8K8	*LINF 320046200*	60S ribosomal protein L2—putative	48.62	46.96	1.66
A4I9N5	*LINF 340012100*	26S proteasome regulatory subunit RPN11	44.87	43.23	1.64
A4IDK9	*LINF 360030600*	Glyceraldehyde-3-phosphate dehydrogenase	51.9	50.28	1.62
A4IA13	*LINF 340028800*	Asparagine—tRNA ligase	46.57	44.95	1.62
**A4I4C9**	***LINF 290012700***	**Heat shock protein 90—putative**	**46.62**	**45.01**	**1.61**
A4IA34	*LINF 340031800*	Alba—putative	48.1	46.51	1.59
A4HWB9	*LINF 150007100*	60S ribosomal protein L13a—putative	47.3	45.72	1.58
**A4HY43**	***LINF 190006800***	**ADP/ATP translocase**	**46.72**	**45.25**	**1.47**
**A4I7P2**	***LINF 320013000***	**Putative RNA binding protein**	**47.01**	**45.58**	**1.43**
**A4ICN5**	***LINF 360015600***	**40S ribosomal protein S10—putative**	**48.51**	**47.08**	**1.43**
A4I3C8	*LINF 280010400*	40S ribosomal protein S26	47.57	46.15	1.42
A4HUU6	*LINF 110008400*	14-3-3 protein 2—putative	45.68	44.3	1.38
A4I7Q4	*LINF 320014400*	60S ribosomal protein L18a	48.71	47.36	1.35
**A4HX65**	***LINF 170005000***	**Hypothetical protein—conserved**	**46.02**	**44.72**	**1.3**
A4HWR3	*LINF 160006500*	Eukaryotic translation initiation factor 1A—putative	47.27	46	1.27
A4HRG4	*LINF 010009200*	40S ribosomal protein S7	48.84	47.59	1.25
A2CIA0	*LINF 100008300*	Isocitrate dehydrogenase [NADP]	45.5	44.29	1.21
A4I291	*LINF 260020700*	Putative thimet oligopeptidase	44.7	43.57	1.13
A4HVK7	*LINF 130012800*	Hypothetical protein—conserved	45.01	43.91	1.1
A4I048	*LINF 230006100*	GDP-mannose pyrophosphorylase	46.69	45.59	1.11
**A4HZ42**	***LINF 210009700***	**Hypothetical protein—conserved**	**45.55**	**44.49**	**1.06**
E9AHD5	*LINF 270012500*	Hypothetical protein—conserved	46.61	45.62	0.99
A4I977	*LINF 330026600*	Hypothetical protein—conserved	45.05	44.08	0.97
A4ICP1	*LINJ 36 0990*	Putative 40S ribosomal protein S18	46.28	45.33	0.95
A4IB31	*LINF 350018700*	Mitochondrial processing peptidase—beta subunit—putative	44.73	43.8	0.93
**A4I218**	***LINF 260013700***	**40S ribosomal protein S16—putative**	**47.59**	**46.7**	**0.89**
A4HXK6	*LINF 170020200*	Putative translation initiation factor	48.53	47.67	0.86
A4I5F6	*LINF 300018100*	Pyridoxal kinase	47.5	46.64	0.86
A4HX73	*LINF 170005900*	Elongation factor 1-alpha	47.64	46.8	0.84
A4HUX3	*LINF 110012000*	Aminopeptidase—putative	50.57	49.74	0.83
A4IE56	*LINF 360050900*	Oxidoreductase—putative	45.04	44.22	0.82
A4I154	*LINF 250006300*	Electron transfer flavoprotein subunit beta	51.28	50.46	0.82
A0A381MM20	*LINF 250017900*	ATP synthase subunit beta	52.11	51.3	0.81
A0A6L0Y0Y0	*LINF 350005400*	Pyruvate kinase	52.63	51.83	0.80
A4HRT6	*LINF 030007300*	Putative ribosomal protein L38	49.14	48.35	0.79
A4HYW1	*LINF 200016800*	Cysteine peptidase—Clan CA—family C2—putative	45.84	45.11	0.73
A0A381MCG8	*LINF 110015600*	40S ribosomal protein S5	46.38	45.69	0.69
A4HV26	*LINF 110017900*	40S ribosomal protein S15A—putative	45.56	44.91	0.65
A4HX92	*LINF 170008800*	Cystathionine beta-synthase	49.33	48.68	0.65
A4I2G1	*LINF 260028800*	60S ribosomal protein L35—putative	47.8	47.18	0.62
E9AHK3	*LINF 300041000*	S-adenosylmethionine synthase	47.24	46.67	0.57
A4HT78	*LINJ 07 0550*	60S ribosomal protein L7a	46.98	46.41	0.57
A4HUJ7	*LINF 100015200*	Nuclear transport factor 2—putative	51.02	50.46	0.56
A4I067	*LINF 230008000*	Pteridine reductase 1	48.78	48.23	0.55
A4I5W4	*LINF 300033900*	Hypothetical protein—conserved	46.7	46.16	0.54
A4I114	*LINF 240026700*	60S ribosomal protein L26—putative	48.33	47.82	0.51
A4I3H3	*LINF 280015200*	40S ribosomal protein S14	45.27	44.79	0.48
**A4I7N0**	***LINF 320010500***	**Profilin**	**46.96**	**46.57**	**0.39**
A4I4E4	*LINF 290014400*	ADP-ribosylation factor-like protein 3A—putative	45.2	44.87	0.33
A4HS39	*LINF 040009500*	Cysteine peptidase—Clan CA—family C2—putative	49.47	49.24	0.23
E9AHM9	*LINF 330009000*	Heat shock protein 83–1	45.27	45.07	0.20
**A4I2Y7**	***LINF 270024900***	**Phosphoenolpyruvate carboxykinase (ATP)**	**45.03**	**44.86**	**0.17**
**A4I9P1**	***LINF 340014000***	**Elongation factor 1-beta**	**43.79**	**43.67**	**0.12**
A4HZI9	*LINF 210028100*	Proteasome subunit alpha type	56.36	56.26	0.10
A4I7R1	*LINF 320015100*	Staphylococcal nuclease homologue /Tudor domain containing protein—putative	59.7	59.61	0.09
A0A381MM90	*LINF 280034800*	Guanine nucleotide-binding protein subunit beta-like protein	44.38	44.35	0.03
**A4I120**	***LINF 240027300***	**3-hydroxy-3-methylglutaryl-CoA synthase—putative**	**48**	**47.98**	**0.02**
A4I6N1	*LINF 310016800*	Biotin/lipoate protein ligase-like protein	43.08	43.06	0.02
**MTX**					
**Accession**	**Gene ID**	**Description**	**T**_**m**_ **(°C)**		***ΔT***_***m***_ **(°C)**
			**+ MTX**	**- MTX**	
A4IB55	*LINF 350020400*	Putative reiske iron-sulfur protein	60.38	46.98	13.4
A4HS42	*LINF 040009700*	60S ribosomal protein L11 (L5. L16)	51.3	45.39	5.91
A4I5G9	*LINF 300019400*	Amidohydrolase- putative	54.1	49.7	4.4
A0A6L0XG31	*LINF 260006600*	60S ribosomal protein L7—putative	49.5	45.74	3.76
A4I1F4	*LINF 250017300*	Aldehyde dehydrogenase	59.59	56.21	3.38
A4I067	*LINF 230008000*	Pteridine reductase 1	51.57	48.23	3.34
A4HT63	*LINF 070005600*	Cytochrome c1—heme protein—mitochondrial—putative	46.54	43.42	3.12
A4HTZ8	*LINF 090013900*	Oligopeptidase b	54.75	51.73	3.02
A0A6L0Y0Y0	*LINF 350005400*	Pyruvate kinase	54.57	51.81	2.76
A4I291	*LINF 260020700*	Putative thimet oligopeptidase	46.69	43.99	2.7
A4HW98	*LINF 020005100*	Histone H4	49.82	47.28	2.54
A4HVN8	*LINF 130016000*	Leucyl-tRNA synthetase	47.87	45.39	2.48
A4I5D2	*LINF 300015700*	Ubiquitin conjugation factor E4 B—putative	48.48	46.13	2.35
A4HZB2	*LINF 210007900*	Hexokinase—putative	51.94	49.74	2.2
Q95NF5	*LINF 150019000*	Tryparedoxin peroxidase	49.76	47.58	2.18
A4I931	*LINF 330024300*	3-ketoacyl-CoA reductase—putative	44.72	42.56	2.16
E9AHH9	*LINF 280015700*	Putative ribosomal protein S20	45.77	43.64	2.13
**A4HWB9**	***LINF 340014400***	**60S ribosomal protein L13a—putative**	**47.67**	**45.68**	**1.99**
A4HVY8	*LINF 140009900*	N-terminal conserved domain of Nudc./CS domain containing protein—putative	46.45	44.62	1.83
**Q9N9V3**	***LINF 040012600***	**Putative ribosomal protein L10**	**48.82**	**47.05**	**1.77**
A0A6L0XZX8	*LINF 350009300*	40S ribosomal protein S3a	47.51	45.83	1.68
A4I114	*LINF 240026700*	60S ribosomal protein L26—putative	48.17	46.49	1.68
**A4ICM4**	***LINF 360016500***	**Putative ribosomal protein L24**	**48.69**	**47.25**	**1.44**
A4HVI5	*LINF 130010500*	60S ribosomal protein L18—putative	48.47	47.04	1.43
A4HZF8	*LINF 210024300*	Putative RNA helicase	47.21	45.83	1.38
A4HXG5	*LINF 170015800*	META domain containing protein	45.13	43.79	1.34
A4HUB4	*LINF 100005600*	Putative ribosomal protein l35a	47.82	46.49	1.33
A4IB38	*LINF 350019600*	Hypothetical protein—conserved	45.75	44.43	1.32
A4HRG4	*LINF 010009200*	40S ribosomal protein S7	48.99	47.68	1.31
**A4I5W4**	***LINF 300033900***	**Hypothetical protein—conserved**	**47.07**	**45.85**	**1.22**
A4IB31	*LINF 350018700*	Mitochondrial processing peptidase -beta subunit—putative	44.93	43.74	1.19
A4IDS4	*LINF 360060700*	40S ribosomal protein SA	45.96	44.81	1.15
A4I3C8	*LINF 280010400*	40S ribosomal protein S26	47.26	46.15	1.11
A0A381MG06	*LINF 190005200*	Histone H2B	49.97	48.92	1.05
A4HY61	*LINF 190008800*	40S ribosomal protein S13—putative	48.37	47.33	1.04
A4ICV5	*LINF 360008400*	Proteasome subunit beta	55.49	54.45	1.04
A4I7P2	*LINF 320013000*	Putative RNA binding protein	46.99	45.96	1.03
**A4I7K4**	***LINF 320009100***	**ATP-dependent RNA helicase**	**47.48**	**46.48**	**1**
A4HV26	*LINF 110017900*	40S ribosomal protein S15A—putative	45.91	44.91	1
**A4IDK9**	***LINF 360030600***	**Glyceraldehyde-3-phosphate dehydrogenase**	**51.08**	**50.09**	**0.99**
A0A6L0X791	*LINF 170007100*	Elongation factor 1-alpha	47.74	46.77	0.97
E9AGK4	*LINF 130022300*	Pyrroline-5-carboxylate reductase	51.21	50.24	0.97
A4IA13	*LINJ 34 2110*	Asparagine-tRNA ligase	45.92	44.99	0.93
A0A6L0WKL6	*LINF 100016800*	Histone H3—putative	49.12	48.2	0.92
A0A381MCG8	*LINF 110015600*	40S ribosomal protein S5	46.44	45.64	0.80
**E9AG68**	***LINF 060011400***	**60S ribosomal protein L23a—putative**	**47.42**	**46.67**	**0.75**
A4HYX4	*LINF 200018300*	Putative small myristoylated protein-1	45.6	44.88	0.72
A0A6L0XQ48	*LINF 330024700*	Peptidyl-prolyl cis-trans isomerase	44.52	43.81	0.71
A4I6N1	*LINF 310016800*	Biotin/lipoate protein ligase-like protein	43.92	43.22	0.70
A4ID08	*LINF 360047700*	Eukaryotic translation initiation factor 3 subunit I	48.09	47.44	0.65
A4HZ42	*LINF 210009700*	Hypothetical protein—conserved	45.27	44.64	0.63
A4HZ33	*LINF 210008800*	Mitochondrial processing peptidase alpha subunit -putative	44.31	43.68	0.63
A4I212	*LINF 260013100*	Glutathione peroxidase	45.16	44.54	0.62
A0A6L0XIU2	*LINF 290021400*	Nodulin-like—putative	45.32	44.71	0.61
**A2CIA0**	***LINF 100008300***	**Isocitrate dehydrogenase [NADP]**	**44.85**	**44.25**	**0.60**
A4HVQ1	*LINJ 13 1130*	Putative 40S ribosomal protein S4	46.96	46.36	0.60
A4I8K8	*LINF 320046200*	60S ribosomal protein L2—putative	47.64	47.05	0.59
E9AGQ8	*LINF 190005400*	40S ribosomal protein S2	47.27	46.69	0.58
A4I5Y5	*LINF 300036000*	Eukaryotic translation initiation factor 3 subunit 7-like protein	44.71	44.13	0.58
Q9BHZ6	*LINF 090016000*	Elongation factor-1 gamma	45.55	44.99	0.56
E9AHK3	*LINF 300041000*	S-adenosylmethionine synthase	47.22	46.72	0.5
A4I4F2	*LINF 290015200*	5-histidylcysteine sulfoxide synthase	43.55	43.09	0.46
A4IDL3	*LINF 360031300*	Methyltransferase	46.44	46.02	0.42
A4HRP2	*LINF 020009800*	Voltage-dependent anion-selective channel-putative	48.47	48.12	0.35
A4ID05	*LINF 360048000*	Adenosylhomocysteinase	51.22	50.91	0.31
**A4I1R2**	***LINF 250028300***	**Succinate-CoA ligase [ADP-forming] subunit alpha**	**45.44**	**45.16**	**0.28**
A4I120	*LINF 240027300*	3-hydroxy-3-methylglutaryl-CoA synthase- putative	48.54	48.27	0.27
**A4HUX3**	***LINF 110012000***	**Aminopeptidase—putative**	**49.4**	**49.15**	**0.25**
**E9AHM9**	***LINF 330009000***	**Heat shock protein 83–17**	**45.72**	**45.5**	**0.22**
A0A381MM90	*LINF 280034800*	Guanine nucleotide-binding protein subunit beta-like protein	44.55	44.35	0.20
A4HRT1	*LINF 030006800*	Multifunctional fusion protein	44.09	43.93	0.16
**A4IBL4**	***LINF 350037700***	**Cystathione gamma lyase—putative**	**44.64**	**44.48**	**0.16**
A4HZS1	*LINF 220009800*	40S ribosomal protein S15—putative	47.19	47.03	0.16
A4I5F6	*LINF 300018100*	Pyridoxal kinase	46.94	46.78	0.16
A4IA34	*LINF 340031800*	Alba—putative	47.15	47	0.15
A4I0C0	*LINF 230014600*	3-ketoacyl-CoA thiolase-putative	49.13	48.99	0.14
Q9N9V8	*LINF 300042300*	Ribosomal protein L15	46.75	46.61	0.14
**A4I4E4**	***LINF 290014400***	**ADP-ribosylation factor-like protein 3A-putative**	**44.58**	**44.46**	**0.12**
A4IBE2	*LINF 350032700*	Galactokinase-like protein	46.14	46.02	0.12
A4I6Z4	*LINF 310029700*	Prostaglandin f2-alpha synthase/D-arabinose dehydrogenase	43.59	43.48	0.11
A4I3X3	*LINF 280031300*	Dihydrolipoamide acetyltransferase component of pyruvate dehydrogenase complex	47.76	47.65	0.11
A4HT92	*LINF 070012400*	40S ribosomal protein S9—putative	46.5	46.42	0.08
A4I993	*LINF 330034600*	Carboxypeptidase—putative	48.36	48.34	0.02
**A4I5X6**	***LINF 300035000***	**Glyceraldehyde-3-phosphate dehydrogenase**	**46.11**	**46.09**	**0.02**

**Fig 5:**
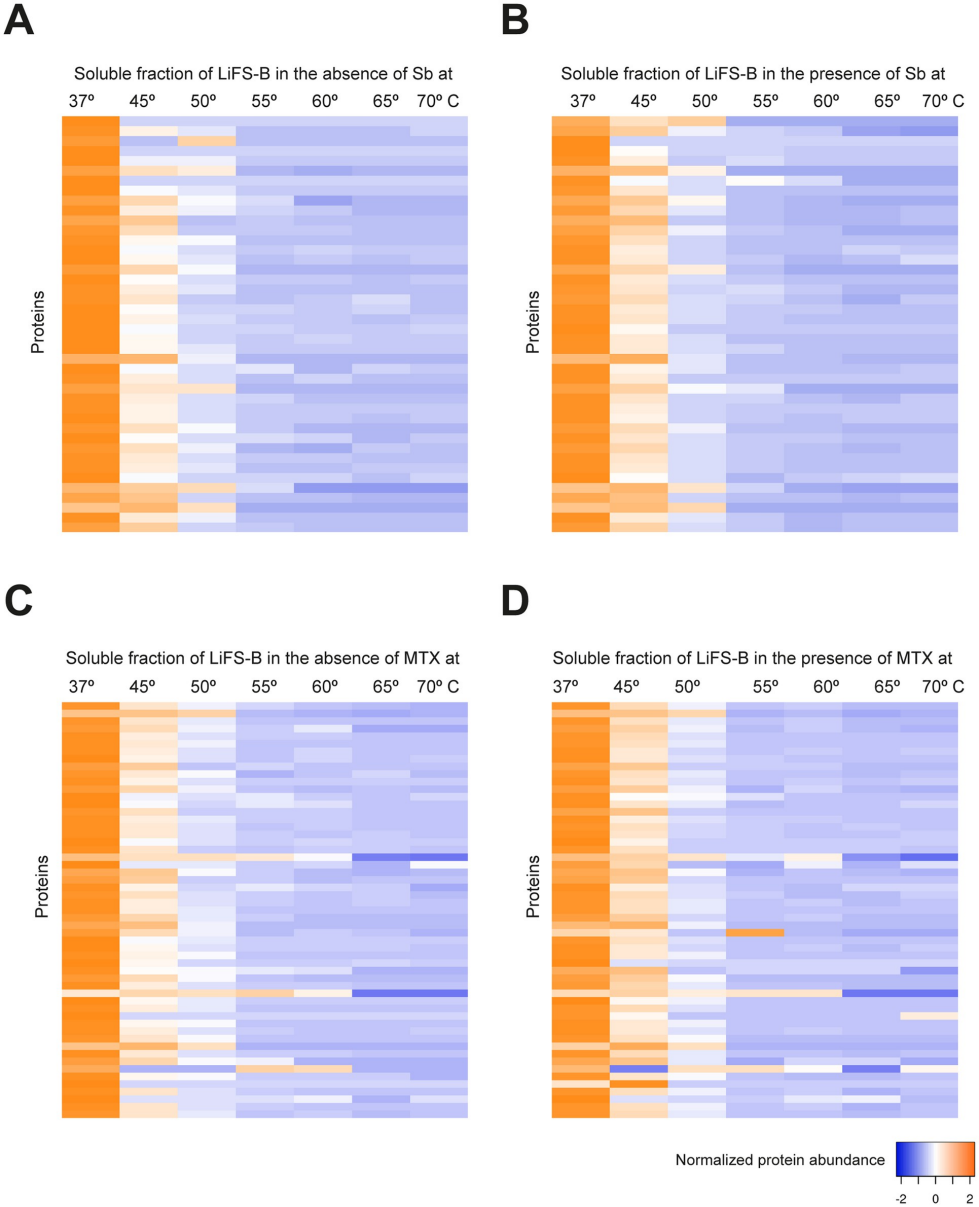
Heat map representation (row Z-score) of the general thermal stability of *Li*FS-B soluble protein cell extracts. Normalized protein abundance of *Li*FS-B proteins for which full melting curves were acquired in the absence (**A**) or in the presence (**B**) of 100 μM Sb (42 proteins) and in the absence (**C**) or in the presence (**D**) of 100 μM MTX (55 proteins). Color range depicts the relative protein abundance of the soluble fractions at different temperatures. Heat maps were generated through the Heat mapper webserver (www.heatmapper.ca/expression) using its protein expression plugin with average linkage as clustering method applied to rows and Euclidean as distance measurement method. Of note, within the two field strains, 20 proteins were identified as shared following exposure to Sb. These shared proteins encompass ribosomal proteins, elongation factors, and heat shock proteins. Additionally, 14 proteins were recognized as common to both strains subsequent to their interaction with MTX, highlighting a prevalence of ribosomal proteins and those associated with the parasite’s cellular respiration.

Next, we conducted an evaluation of the proteomic profile in the antimony-resistant clinical isolate *Li*FS-B, both in the presence and absence of Sb. Among the 1193 soluble proteins identified (S1 Data), 42 exhibited a pattern that allowed us to calculate melting curves (Fig 5A and 5B). Compared to *Li*FS-A, the Sb-resistant strain showed a general decrease in protein-thermal stabilization, with 76 fewer proteins exhibiting temperature variations suitable for melting curve calculations (118 in *Li*FS-A versus 42 in *Li*FS-B), which further confirms a decreased interaction of Sb with its proteome. Within these 42 proteins, particularly those exhibiting the highest *ΔTm*, we identified several key proteins: an alanine-tRNA ligase (A4I013), two ribosomal proteins–the 60S acidic ribosomal protein P0 and the 40S ribosomal protein S24 (A4I2U1 and A4ID74, respectively)–, a putative 60S ribosomal protein (L10A), a putative ATP synthase F1 subunit protein (A4HZI3), a eukaryotic translation initiation factor (A4I5Y5), and an uncharacterized protein (A4HZ42) (Table 3). To elucidate the potential function of the uncharacterized protein and its involvement in Sb resistance, we employed databases such as PantherDB (http://www.pantherdb.org/, accessed on 20 June 2023), InterPro (https://www.ebi.ac.uk/interpro/, accessed on 20 June 2023), and Uniprot (https://www.uniprot.org/, accessed on 30 January 2024). This search revealed a possible orthologous relationship (92.33% identity) between our uncharacterized protein and a NTF2 (Nuclear transport factor 2) domain-containing protein found in the same chromosome of *L*. *major* (LMJF_21_0430, accession number Q4QCH42), potentially belonging to Ras-GTPase-activating protein-binding.

**Table 3:** Summary of proteins identified in Sb-treated and MTX-treated *Li*FS-B, demonstrating a positive temperature shift. Proteins that are common between the strains *Li*FS-B and *Li*FS-A are highlighted in bold for easy identification.

**Sb**					
**Accession**	**Gene ID**	**Description**	***Tm* (°C)**		***ΔTm* (°C)**
			**+ Sb**	**- Sb**	
A4I013	*LINF 220021600*	Alanine tRNA ligase	49.65	40.39	9.26
A4I2U1	*LINF 270020500*	60S acidic ribosomal protein P0	48.56	43.75	4.81
A4ID74	*LINF 360036600*	40S ribosomal protein S24	40.36	37.07	3.29
A4HXT8	*LINF 360046500*	Putative 60S ribosomal protein L10A	43.41	40.37	3.04
**A4HZ42**	***LINF 210009700***	**Uncharacterized protein**	**44.87**	**42.1**	**2.77**
A4HZI3	*LINF 210027500*	Putative ATP synthase F1 subunit gamma protein	49.76	47.08	2.68
A4I5Y5	*LINF 300036000*	Eukaryotic translation initiation factor 3 subunit 7-like protein	42.62	40.39	2.23
**A4ICN5**	***LINF 360015600***	**Putative 40s ribosomal protein S10**	**44.47**	**42.5**	**1.97**
A4I0C2	*LINF 230014800*	Acetyl coenzyme A synthetase	48.69	46.87	1.82
**A4HZF8**	***LINF 210024300***	**ATP-dependent RNA helicase SUB2—putative**	**45.77**	**44.11**	**1.66**
A4I1R2	*LINF 250028300*	Succinate CoA ligase [ADP-forming] subunit alpha	47.27	45.67	1.6
A4I1Z8	*LINF 260011600*	protein disulfide isomerase—putative	46.5	44.91	1.59
**E9AG68**	***LINF 060011300***	**Putative 60S ribosomal protein L23a**	**44.57**	**43.04**	**1.53**
A4I5C0	*LINF 300014400*	Putative Adenosine kinase	43.7	42.38	1.32
**A4I9P1**	***LINF 340014000***	**Elongation factor 1-beta**	**44.56**	**43.32**	**1.24**
A4HRR9	*LINF 270033860*	Dipeptylcarboxypeptidase	47.18	45.96	1.22
**A4I7P2**	***LINF 320013000***	**Putative RNA binding protein**	**43.99**	**42.9**	**1.09**
A4I9G9	*LINF 340005000*	Short chain dehydrogenase putative	44.47	43.38	1.09
A4I931	*LINF 330024300*	3-ketoacyl-CoA reductase putative	45.8	44.75	1.05
**A4HVS0**	***LINF 130021300***	**Ubiquitin conjugating enzyme-like protein**	**44.69**	**43.64**	**1.05**
A4HW62	*LINF 140018000*	Enolase	44.38	43.37	1.01
**A4I218**	***LINF 260013700***	**Putative 40s ribosomal protein s16**	**43.72**	**42.82**	**0.90**
**A4I2Y7**	***LINF 270024900***	**Glycosomal phosphoenolpyruvate carboxykinase. putative**	**43.97**	**43.11**	**0.86**
A4HWZ0	*LINF 160015100*	Sucrose phosphate synthase like protein	43.78	42.93	0.85
**A4I120**	***LINF 240027300***	**3-hydrox-3-methylglutaryl-CoA synthase—putative**	**49.77**	**49.01**	**0.76**
**A4HUB4**	***LINF 100005600***	**Putative Ribosomal protein L35A**	**43.59**	**42.83**	**0.76**
A0A0S2UQ61	*LINF 280034700*	Activated protein kinase C receptor	44.21	43.51	0.70
**A4I7N0**	***LINF 320010500***	**Profilin**	**46.94**	**46.39**	**0.55**
A4HTP4	*LINF 080016000*	Stressinduced protein sti1	44.69	44.15	0.54
**A4I4W0**	***LINF 290032300***	**Putative 60S ribosomal protein L13**	**43.96**	**43.47**	**0.49**
A4HSP6	*LINF 060005200*	WD domain G-beta repeat putative	43.72	43.32	0.40
**A4HWV9**	***LINF 160010900***	**Core histone H2A/H2B/H3/H4/Histone-like transcription factor (CBF/NF-Y) and archaeal histone—putative**	**45.56**	**45.18**	**0.38**
**A4I4C9**	***LINF 290012700***	**Heat shock protein 90 putative**	**44.26**	**43.96**	**0.30**
A4IBL4	*LINF 350037700*	Putative cystathione gamma lyase	45.82	45.53	0.29
**A4I5X6**	***LINF 300035000***	**Glyceraldehyde-3-phosphate dehydrogenase**	**44.25**	**43.99**	**0.26**
**A4HY43**	***LINF 190006800***	**ADP—ATP carrier protein 1**	**43.94**	**43.78**	**0.16**
**A4HX65**	***LINF 170005000***	**Uncharacterized protein**	**43.19**	**43.04**	**0.15**
**A4HZB2**	***LINF 210007900***	**Hexokinase—putative**	**51.84**	**51.77**	**0.07**
A4ICK8	*LINF 360018400*	Fructose bisphosphate aldolase	46.7	46.65	0.05
A4HW34	*LINF 140015200*	Glutathione synthetase	50.28	50.23	0.05
**A4I7K4**	***LINF 320009100***	**Putative ATP-dependent RNA helicase**	**44.09**	**44.05**	**0.04**
A4I341	*LINF 270032200*	Putative heat shock protein DnaJ	45.94	45.9	0.04
MTX					
**Accession**	**Gene ID**	**Description**	**Tm (°C)**		**ΔTm (°C)**
			**+ MTX**	**- MTX**	
A4I1G1	*LINF 250018000*	ATP synthase subunit beta	50.14	33.41	16.73
A4HXP7	*LINF 180007100*	Activator of Hsp90 ATPase	59.62	47.24	12.38
A4I088	*LINF 230010200*	SNF1	49.17	40.46	8.71
A4HRH2	*LINF 010010000*	Putative long-chain-fatty-acid-CoA ligase	44.83	40.46	4.37
A4I048	*LINF 230006100*	GDP-mannose pyrophosphorylase	46.45	42.94	3.51
A4HXB7	*LINF 170010700*	Hypothetical protein—conserved	44.58	42.07	2.51
**Q9N9V3**	***LINF 040012600***	**Putative ribosomal protein L10**	**45.08**	**42.75**	**2.33**
**A4I5W4**	***LINF 300033900***	**Hypothetical protein—conserved**	**49.85**	**47.7**	**2.15**
A4ICN5	*LINF 360015700*	Putative 40s ribosomal protein S10	44.61	42.5	2.11
E9AHB0	*LINF 260006700*	Putative 60s ribosomal protein l7	45.56	43.67	1.89
A4I5C0	*LINF 300014400*	Putative Adenosine kinase	43.99	42.42	1.57
A4I2S6	*LINF 270019000*	Putative T-complex protein 1. beta subunit	45.04	43.53	1.51
A4I218	*LINF 260013800*	Putative 40s ribosomal protein s16	44.97	43.51	1.46
A4HUU6	*LINF 110008400*	Putative 14-3-3 protein	46.19	44.81	1.38
A4HVS0	*LINF 130021300*	Ubiquitin-conjugating enzyme-like protein	44.76	43.39	1.37
**A4HWB9**	***LINF 340014400***	**Putative 60S ribosomal protein L13a**	**45.22**	**43.92**	**1.3**
E9AHK7	*LINF 310006400*	Putative ubiquitin hydrolase	44.65	43.44	1.21
**A4I5X6**	***LINF 300035000***	**Glyceraldehyde-3-phosphate dehydrogenase**	**44.85**	**43.74**	**1.11**
A4HTP4	*LINF 080016000*	Stress-induced protein sti1	45.41	44.31	1.10
**A4IDK9**	***LINF 360030600***	**Glyceraldehyde-3-phosphate dehydrogenase**	**46.84**	**45.76**	**1.08**
**A4ICM4**	***LINF 360016500***	**Putative ribosomal protein l24**	**44.87**	**43.79**	**1.08**
**E9AG68**	***LINF 060011400***	**Putative 60S ribosomal protein L23a**	**43.95**	**43.04**	**0.91**
A4I4W0	*LINF 290032300*	Putative 60S ribosomal protein L13	44.36	43.48	0.88
A4I3V7	*LINF 280029400*	Putative glycosomal membrane protein	44.67	43.83	0.84
**A4HUX3**	***LINF 110012000***	**Putative aminopeptidase**	**46.85**	**46.11**	**0.74**
A4I9V2	*LINF 340021000*	N-terminal region of Chorein	45.32	44.6	0.72
**A4I7K4**	***LINF 320009100***	**ATP-dependent RNA helicase**	**44.75**	**44.08**	**0.67**
**A2CIA0**	***LINF 100008300***	**Isocitrate dehydrogenase [NADP]**	**44.06**	**43.39**	**0.67**
**A4I1R2**	***LINF 250028300***	**Succinate-CoA ligase**	**46.24**	**45.61**	**0.63**
A4HVE5	*LINF 130005800*	Putative carboxypeptidase	59.82	59.19	0.63
A4HYW1	*LINF 200016800*	Putative calpain-like cysteine peptidase	47.16	46.55	0.61
Q2PDB9	*LINF 180021400*	Inosine-uridine preferring nucleoside hydrolase	61.09	60.51	0.58
A4IC14	*LINF 350053300*	Cyclophilin 40	43.98	43.42	0.56
A4HW83	*LINF 140020100*	Putative tyrosyl-tRNA synthetase	44.08	43.57	0.51
A4I341	*LINF 270032200*	Putative heat shock protein DnaJ	46.2	45.72	0.48
A4IA31	*LINF 340031400*	Uncharacterized protein	45.92	45.46	0.46
A4HU72	*LINF 090022600*	Cytochrome b5-like protein	45.92	45.46	0.46
A4HX73	*LINF 170007200*	Elongation factor 1-alpha	44.91	44.49	0.42
A4HX65	*LINF 170005000*	Uncharacterized protein	42.89	42.49	0.39
A4I7I7	*LINF 320007400*	Putative dynein light chain. flagellar outer arm	44.95	44.57	0.38
A4I9B4	*LINF 330036700*	Putative translation initiation factor IF-2	38.42	38.11	0.31
A0A0S2UQ61	*LINF 280034700*	Activated protein kinase C receptor	44.07	43.78	0.29
A4HVL6	*LINF 130013700*	Uncharacterized protein	47.44	47.17	0.27
A4I8S7	*LINF 330013000*	Paraflagellar rod component—putative	48.51	48.25	0.26
A4HY43	*LINF 190006800*	ADP-ATP carrier protein 1	44.33	44.08	0.25
**E9AHM9**	***LINF 330009000***	**Heat shock protein 83–17**	**45.29**	**45.06**	**0.23**
A4HZI4	*LINF 350025100*	40S ribosomal protein S6	44.98	44.75	0.23
A4I0M7	*LINF 240005200*	Ribosomal protein L22p/L17e—putative	46.02	45.82	0.20
A4ID12	*LINF 360047300*	Putative glycyl tRNA synthetase	43.92	43.78	0.14
**A4I4E4**	***LINF 290014400***	**ADP-ribosylation factor-like protein 3A**	**45.14**	**45.04**	**0.10**
A2CIN2	*LINF 290027200*	Fumarate hydratase	44.54	44.45	0.09
A4HWZ0	*LINF 160015100*	Sucrose phosphate synthase-like protein	43	42.93	0.07
A4HW34	*LINF 140015200*	Glutathione synthetase	50.27	50.23	0.04
A4I4C9	*LINF 290012700*	Heat shock protein 90—putative	43.57	43.55	0.02
**A4IBL4**	***LINF 350037700***	**Putative cystathione gamma lyase**	**45.12**	**45.1**	**0.02**

Finally, we evaluated the proteomic meltome profile in the *Li*FS-B strain in the absence and presence of MTX. Among the 1112 soluble proteins identified (S1 Data), melting curves were determined for 55 of them. Like the other drug analyzed, we observed protein stabilization at low temperatures, both with and without the presence of MTX (Fig 5C and 5D). Table 3 summarizes the proteins identified, including ATP synthase subunit beta (A4I1G1), an activator of Hsp90 ATPase (A4HXP7), a SNF1-related protein kinase (A4I088), a putative long-chain fatty acid (A4HRH2), a GDP-mannose pyrophosphorylase (A4I048), two conserved hypothetical proteins (A4HXB7 and A4I5W4), and two ribosomal proteins (L10 and S10).

## Discussion

The primary challenge in immunosuppressed patients with VL remains the inadequate effectiveness of antileishmanial treatments and the heightened risk of relapses [31,32]. This predicament is further exacerbated by the escalating emergence of drug-resistant strains in *Leishmania* parasites [33]. A deficient immune response can largely permit the outgrowth of persisters or other *Leishmania* variants that exhibit intermediate resistance levels. Of note, due to its genomic plasticity–coupled to the shared use of antileishmanials in animals and humans–, for many *Leishmania* infections, drug resistant parasites are likely present by the time chemotherapy starts [34]. Current research on drug resistance in *Leishmania* during treatment has mainly focused on the interaction between the parasite and antileishmanials, frequently ignoring the direct impact of other drugs such as immunosuppressants on *Leishmania* evolution. This study uncovers, for the first time, cross-resistance between MTX and Sb in clinical *L*. *infantum* isolates. This finding is significant as it may compromise treatment efficacy in immunosuppressed patients and contribute to the spread of drug-resistant parasites.

First, we assessed the responsiveness of both isolates to Sb and MTX, focusing on their capability to regulate ROS levels. *Li*FS-A exhibited a susceptibility to Sb and demonstrated an increased accumulation of ROS when exposed to this drug. This could be explained by the fact that the trypanothione/trypanothione reductase (TR) system, essential for the parasite’s oxidoreductive balance, is disrupted by trivalent Sb. This disruption causes a rapid efflux of T[SH]2 and glutathione and leads to apoptosis by increasing ROS and intracellular Ca^2+^ levels [35,36]. On the other hand, isolate *Li*FS-B was able to control ROS levels following MTX and Sb exposure, which correlated with a drug-resistant phenotype against both drugs. Of note, it has been observed that *Leishmania* strains recovered from immunosuppressed patients exhibit decreased sensitivity to Sb-based treatments [37]. Notably, most cases of secondary treatment failure with Sb occur in immunosuppressed patients due to their diminished immune response, which promotes parasite multiplication and hampers the efficacy of antimonial drugs, facilitating resistance development and subsequent relapses in leishmaniasis after treatment [37,38].

Next, we evaluated the expression of key genes involved in resistance against Sb and MTX. One of the most significant findings of this study is the clear evidence that pre-exposure to Sb results in a notable increase in the EC_50_ against MTX, and conversely, pre-exposure to MTX leads to a similar increase in the EC_50_ against Sb. Previous *in vitro* studies have demonstrated that exposure of parasites to Sb can lead to the co-amplification of the *mrpA* and *ptr1* coding genes. These genes are in proximity (chromosome 23), and their amplification can occur through rearrangements within the same intergenic regions [34]. Our analyses of field isolates did not reveal overexpression of either of these two genes. However, we were able to identify a clear upregulation of *dhfr* in both MTX-resistant isolates. This finding is consistent with the previous study conducted by Rastrojo and colleagues, where they demonstrated the overexpression of the *dhfr* transcript gene in a Sb-resistant strain of *L*. *donovani* [39]. While some studies have reported the upregulation of *dhfr* in Sb-resistant strains, indicating its potential involvement in resistance, other studies have not observed significant changes in *dhfr* expression levels. It is important to note that drug resistance in *Leishmania* (as illustrated in this work) is multifactorial and can involve a combination of mechanisms, including alterations in drug transporters, drug metabolism, DNA repair mechanisms, and drug target modifications. Further research and investigation are required to fully elucidate the potential role of DHFR in Sb resistance and to better understand its significance in the overall resistance mechanism.

Finally, to better understand the interactions of Sb and MTX with *Leishmania* proteins in these two clinical isolates–as well as the potential mechanisms of cross-resistance–, we used a TPP-TR recently implemented for *Leishmania* parasites [25]. TPP analysis conducted in the presence of Sb indicated that proteins in the Sb-sensitive strain *Li*FS-A were associated with the activation of the mitochondrial respiratory chain. Within this group of proteins, we identified an NADH oxidase protein that it is known to increase its expression following exposure to Sb, resulting in an excessive production of superoxide [40]. Additionally, a mitochondrial cytochrome C1 protein was discovered, which, upon binding to Sb, could disrupt ATP synthesis, ultimately leading to the elimination of the parasites. Furthermore, we observed a highly thermally stable iron-sulfur protein that is potentially associated with the inhibition of thiol metabolism. On the other hand, the analysis of the meltome of the Sb-resistant isolate (*Li*FS-B) revealed significant stabilization of an alanine-tRNA ligase in the presence of Sb, along with three ribosomal proteins. These four proteins play a crucial role in ribosomal biogenesis and protein synthesis. During the late stage of promastigote differentiation, there is typically a metabolic stabilization accompanied by a decrease in the abundance of ribosomal proteins and tRNA synthetases [41]. However, variations in protein abundance could indicate a heightened metabolic activity and functional adaptation to external factors [42], such as drug pressure. Additionally, ribosomal proteins, along with translational proteins and others, contribute to the proliferation of promastigotes, allowing them to evade the host immune response and increasing their virulence [43], which is supported by findings showing that Sb-resistant field strains display increased virulence [44]. It is important to note that using the TPP-TR approach we cannot identify any direct interaction of MRPA with Sb, as this must be conjugated with thiols to bid the ABC transporter [9]. However, we were able to prove that *mrpA* expression levels are higher in the *Li*FS-B isolate.

In TPP experiments with MTX, we observed a significant increase in various proteins involved in mitochondrial processes in both field isolates. This heightened activity could help in detoxifying the effects of MTX, maintaining cellular homeostasis, and potentially activating compensatory pathways that mitigate MTX’s impact. In this way, *Li*FS-A’s meltome in the presence of MTX displayed an enrichment in both cytochrome c reductase activity and translation. By increasing cytochrome c reductase activity, *Leishmania* might improve the efficiency of its mitochondrial electron transport chain, maintaining ATP production and protecting the mitochondria from damage caused by ROS [45]. Enhanced translation in *Leishmania* in response to MTX exposure might be a compensatory mechanism to increase the production of proteins necessary for DNA repair, detoxification, and stress response. On the other hand, MTX induced enrichment in carboxylic acid metabolism proteins in *Li*FS-B’s meltome. This observation aligns with various prior studies which have identified that modifications in carboxylic acid metabolism, through either gene amplification or changes in enzyme functionality, can contribute to the development of MTX resistance in *Leishmania* [46,47]. Our analyses also pinpointed the interaction between PTR1 and MTX in *Li*FS-A. MTX competitively inhibits DHFR, which is responsible for converting dihydrofolate (DHF) to tetrahydrofolate (THF), an essential cofactor in the synthesis of nucleotides. However, PTR1 can convert DHF back to THF, thus bypassing the inhibitory action of MTX on DHFR [22,48]. In addition, we observed higher thermal stabilization of other important proteins, including an ATP synthase subunit beta and an activator of HSP90 ATPase. These findings lead us to hypothesize that there is an increase in the proton gradient during the entry of MTX into the parasites via FBT transport, which may explain the increased stabilization of ATP synthase in at least one of the resistant isolates. Markedly, the observed increase in these chaperones may be linked to an adaptation to stress by promoting protein folding and stability of certain target proteins involved in MTX metabolism, transport, or detoxification pathways.

In summary, our study highlights the potential risk of Sb-MTX cross-resistance selection when *L*. *infantum* parasites are exposed to either of these drugs. This finding may help explain the relapses of visceral leishmaniasis observed in immunosuppressed patients treated with MTX. Importantly, this new knowledge has the potential to inform the development of more tailored immunosuppression regimens, thereby reducing the risk of selecting and spreading drug-resistant parasites, particularly in endemic areas. Additionally, we have provided a comprehensive list of potential Sb- and MTX-interacting proteins and pathways that could be further explored as targets for therapeutic interventions and as biomarkers of drug resistance in future studies.

## Supporting information

S1 Fig Normalized mRNA expression levels of *mrpA*, *ptr1*, and *ass* in *Li*TW non-exposed (-) and pre-exposed (+) to MTX EC_90_. mRNA expression levels of H-locus genes *mrpA* **(A)**, *ptr1* **(B)**, and *ass* **(C)** were determined by quantitative real-time RT-PCR and normalized using *gapdh* as housekeeping gene. Results are derived from three biological replicates. Each data point represents the average ± SEM. Differences were statistically evaluated using an unpaired two-tailed t-test. ** p<0.01; *** p<0.001.
(TIF)

S1 Data Proteomic data generated in this study. (XLSX)
