# Supplementary figures and images for: Potential selection of antimony and methotrexate cross-resistance in Leishmania infantum circulating strains
Source: PLoS Negl Trop Dis. 2024 Feb 29;18(2):e0012015. doi: 10.1371/journal.pntd.0012015 (PMC10931519; doi:10.1371/journal.pntd.0012015)

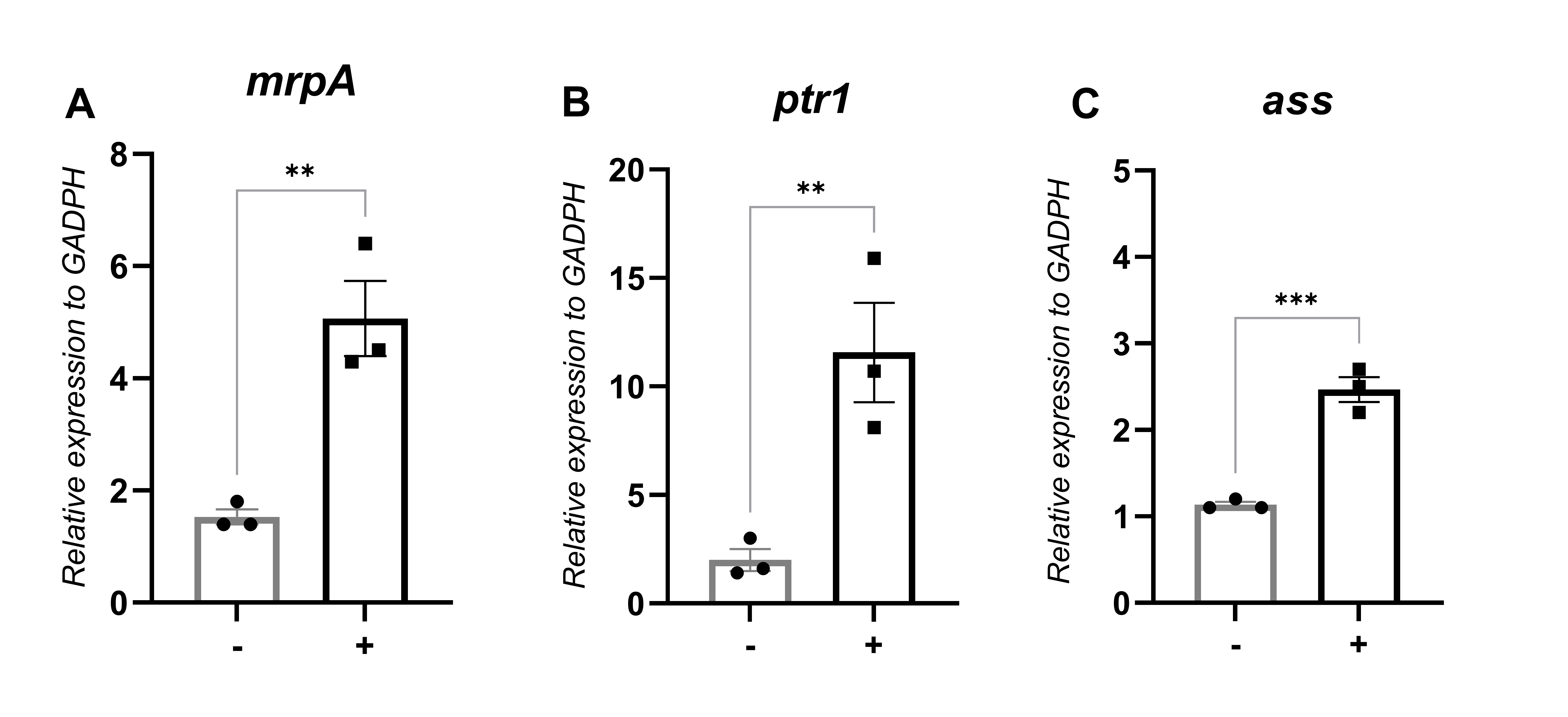

Supplement: S1 Fig — mRNA expression levels of H-locus genes mrpA (A), ptr1 (B), and ass (C) were determined by quantitative real-time RT-PCR and normalized using gapdh as housekeeping gene. Results are derived from three biological replicates. Each data point represents the average ± SEM. Differences were statistically evaluated using an unpaired two-tailed t-test. ** p<0.01; *** p<0.001. (TIF) [file pntd.0012015.s001.tif]
